# Postmating reproductive barriers contribute to the incipient sexual isolation of the United States and Caribbean *Drosophila melanogaster*

**DOI:** 10.1002/ece3.1596

**Published:** 2015-07-14

**Authors:** Joyce Y Kao, Seana Lymer, Sea H Hwang, Albert Sung, Sergey V Nuzhdin

**Affiliations:** 1 Section of Molecular and Computational Biology, Department of Biology, University of Southern CaliforniaLos Angeles, California, 90089; 2 Department of Biology, New York University29 Washington Pl, New York city, New York, 10003; 3 St. Petersburg State Polytechnical UniversitySt. Petersburg, Russia

**Keywords:** Chase away selection, egg laying, hatchability, remating, sexual conflict, sperm toxicity

## Abstract

The nascent stages of speciation start with the emergence of sexual isolation. Understanding the influence of reproductive barriers in this evolutionary process is an ongoing effort. We present a study of *Drosophila melanogaster* admixed populations from the southeast United States and the Caribbean islands known to be a secondary contact zone of European- and African-derived populations undergoing incipient sexual isolation. The existence of premating reproductive barriers has been previously established, but these types of barriers are not the only source shaping sexual isolation. To assess the influence of postmating barriers, we investigated putative postmating barriers of female remating and egg-laying behavior, as well as hatchability of eggs laid and female longevity after mating. In the central region of our putative hybrid zone of American and Caribbean populations, we observed lower hatchability of eggs laid accompanied by increased resistance to harm after mating to less-related males. These results illustrate that postmating reproductive barriers act alongside premating barriers and genetic admixture such as hybrid incompatibilities and influence early phases of sexual isolation.

## Introduction

Speciation is driven by the evolution of reproductive barriers that reduce gene flow and result in reproductive isolation between populations. These barriers are categorized by the temporal nature of their effect: prezygotic barriers occur before fertilization, and postzygotic barriers occur after fertilization (Coyne and Orr 2004). The latter can be further divided into extrinsic and intrinsic subcategories, depending on whether the barrier interacts with external factors (e.g., environmental, individuals) or internal factors (e.g., genetic incompatibilities), respectively (Seehausen et al. 2014). Speciation involves multiple reproductive barriers of varying effect sizes (Coyne and Orr 2004; Seehausen et al. 2014), and identifying the interaction as well as the strength of reproductive barriers at play is vital to characterizing the process of speciation.

*Drosophila* is particularly well suited to study reproductive barriers because species within this genus are highly variable in degree of reproductive isolation, from non-interbreeding species to hybridizing species (Yukilevich and True 2008b; Bono and Markow 2009). Empirical studies of sexual selection in *D. melanogaster* have investigated the evolution of prezygotic isolation – mate choice, male morphology, and courtship behavior (Hollocher et al. 1997; Yukilevich and True 2008a). Postzygotic barrier mechanisms are also known to have an influence in the *Drosophila* genus, but these studies have been limited to hybridizing species *D. mojavensis/D. arizonae* (Bono and Markow 2009) and *D. melanogaster/D. simulans* (Matute et al., 2014).

Many natural forces influence the development of reproductive barriers; one example is sexual conflict, derived from the competing reproductive interests between males and females (Parker 1979). Males may benefit from overriding the mating preferences evolved by females, and females consequently evolve resistance to these male “coercion” tactics (Holland and Rice 1998). Males are then selected for novel or more exaggerated traits (Parker 1979; Civetta and Singh 1995; Rice 1996; Chapman et al. 2003; Arnqvist and Rowe 2005; Arbuthnott et al. 2014).

In *Drosophila melanogaster*, male sperm consists of accessory gland proteins that reduce female remating rates and increase egg laying (Wolfner 1997; Chapman et al. 2003). Reduced receptivity to remating decreases the females’ opportunity to mate with another male that could result in fitter progeny. Increased egg laying and the trauma from mating reduces female life span (Fowler and Partridge 1989). As a result, females develop resistance to these harmful male traits, and males subsequently evolve new methods to discourage females from mating with other males (Arnqvist and Rowe 2005). This phenomenon of conflict in reproductive optima has been experimentally demonstrated to promote an antagonistic male–female coevolution, which may potentially lead to sexual isolation (Parker 1979; Holland and Rice 1998; Chapman et al. 2003). It has been suggested that females are more resistant to males they have coevolved with (“homotypic”) compared to males they have not coevolved with (“heterotypic”). However, this effect varies across populations, and ecological context appears to be a factor (Arbuthnott et al. 2014).

Furthermore, the evolution of Dobzhansky-Muller incompatibilities (DMIs) between populations is also known to promote speciation. Neutral allelic substitution within a population can be incompatible with loci of a divergent population, and these incompatibilities are thought to be generated by various forms of genomic conflict (Seehausen et al. 2014). Negative epistasis reduces the overall viability and sterility of their hybrids, acting as a powerful force underlying incipient reproductive isolation.

A powerful approach to understanding the strength and dynamics of postzygotic isolation is the study of hybrid zones, regions where divergent populations interbreed and produce offspring (Harrison 1990). A secondary hybrid zone emerges when two allopatric populations interbreed after expansion or migration (Jiggins and Mallet 2000). One striking example of a secondary hybrid zone has been discovered in the Caribbean Islands and the southeastern United States. In this region, two ancestral populations of *D. melanogaster,* originating from west Africa and Europe (Yukilevich et al. 2010; Kao et al. 2015), recently came into secondary contact (Bergland et al. 2014) via two waves of colonization: west African flies migrating to the Caribbean Islands during the transatlantic slave trade 400–500 years ago, and then the European flies arriving to the east coast United States with the agency of European colonists <200 years ago (Capy et al. 1986; Duchen et al. 2013).

Caribbean populations display body size, allozyme frequencies, hydrocarbon composition, and sequence variation similar to those of African populations (Capy et al. 1986; Caracristi and Schlötterer 2003; Yukilevich and True 2008a; Kao et al. 2015). Sequence data also suggest that the United States flies display a higher proportion of African alleles than do European flies, suggesting Caribbean populations as a potential source of African alleles introgression for North America populations (Capy et al. 1986; Caracristi and Schlötterer 2003; Yukilevich and True 2008b; Yukilevich et al. 2010; Kao et al. 2015). Mating preferences and other premating/prezygotic reproductive barriers have been formally treated in this system, showing partial sexual isolation between west African flies and American flies, but not Caribbean flies (Yukilevich and True 2008a,b). Male courtship behavior also differs between American and Caribbean flies (Yukilevich and True 2008a,b). However, the presence of postmating sexual isolation in these American and Caribbean populations remains unexplored.

We have investigated the role of remating, female egg laying, hatchability of laid eggs, and female longevity after mating with different males as putative postmating reproductive barriers. These phenotypes are good candidates for assaying the roles of extrinsic and intrinsic postmating reproductive barriers. We measured each of these phenotypes in females from different locations in the southeastern United States and the Caribbean islands to examine them for geographical patterns. Our study aims to explore the role of postmating reproductive barriers in a *Drosophila melanogaster* secondary contact hybrid zone and to better understand how patterns of postmating barriers reflect the colonization history of fly populations in the area. By looking at these roles, we provide general insight into how these isolating mechanisms arise in a genetically admixed system (Kao et al. 2015).

## Materials and Methodology

### Fly lines and rearing conditions

**Figure 1:**
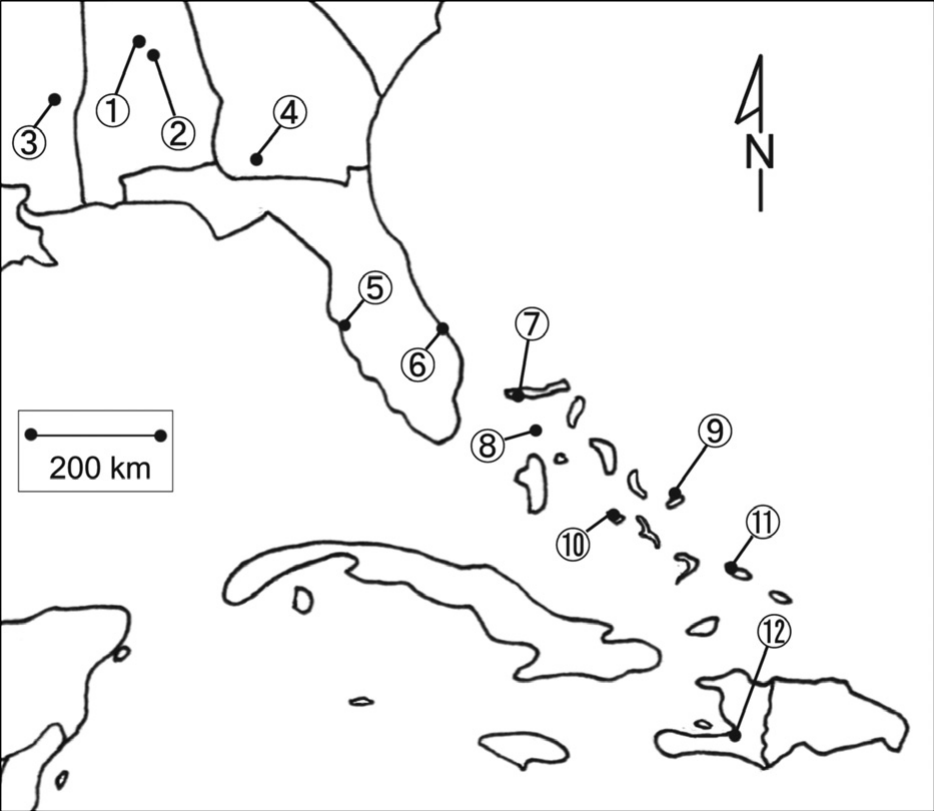
Map of locations used in postmating assays with numbers corresponding to those of Table1.

For our phenotypic assays, we used 23 isofemale lines of *Drosophila melanogaster* collected in the summer of 2004 and 2005 (Yukilevich and True 2008b). The origins of the *Drosophila* are as follows (Table1; Fig.1): Birmingham, AL (lines 1-1 and 1-2); Selba, AL (lines 2-1 and 2-2); Meridian, MS (lines 3-1 and 3-2); Thomasville, GA (lines 4-1 and 4-2); Tampa Bay, FL (lines 5-1 and 5-2); Sebastian, FL (line 6-1); Freeport, Grand Bahamas-west (lines 7-1 and 7-2); Bullock’s Harbor, Berry Islands (lines 8-1 and 8-2); Cockburn Town, San Salvador (lines 9-1 and 9-2); George Town, Exumas (lines 10-1 and 10-2); Mayaguana, Mayaguana (lines 11-1 and 11-2); Port Au Prince, Haiti (lines 12-1 and 12-2). Latitude and longitude coordinates can be found in Yukilevich and True (2008b). All flies were maintained at 25°C in vials on a standard cornmeal diet (recipe available upon request) and entrained under a 12 h light:12 h dark regime.

**Table 1:** Locations, strain names, and line ID numbers of fly lines used in assays

Map Number	Location	Line(s) in order of decreasing latitude (N to S)	Line ID#’s (Yukilevich and True 2008b)
1	Birmingham, AL	1-1 and 1-2	21, 39 and 21, 36
2	Selba, AL	2-1 and 2-2	20, 28 and 20, 17
3	Meridian, MS	3-1 and 3-2	24, 2 and 24, 9
4	Thomasville, GA	4-1 and 4-2	13, 34 and 13, 29
5	Tampa Bay, FL	5-1 and 5-2	4, 12 and 4, 27
6	Sebastian, FL	6-1	28, 8
7	Freeport, Grand Bahamas - West	7-1 and 7-2	33, 16 and 33, 11
8	Bullock’s Harbor, Berry Islands	8-1 and 8-2	40, 23 and 40, 10
9	Cockburn Town, San Salvador	9-1 and 9-2	42, 23 and 42, 20
10	George Town, Exumas	10-1 and 10-2	36, 9 and 36, 12
11	Mayaguana, Mayaguana	11-1 and 11-2	43, 19 and 43, 18
12	Port Au Prince, Haiti	12-1 and 12-2	H, 29 and H, 25

### Egg laying, hatchability, and remating rate assays

Virgin females were collected from all 23 isofemale lines. Male flies up to 1-day old were collected from two lines (lines 1-2 and 11-1) located at polar ends of our geographical study region. We chose these two lines as sources for male flies based on clinal distance as well as maximal difference between courtship profiles and physical characteristics (Yukilevich and True 2008b) to account for female mate preference, which has been previously established (Yukilevich and True 2008a). While using males from two polar locations may prove difficult to distinguish smaller effect sizes of clinal position from line-level variation, we should still be able to reasonably assay big effect sizes from our experimental design. All flies were collected on light CO_2_ anesthesia and aged for 3–4 days before entering our assays. We set up a full factorial experiment in which females from each of the isofemale lines were crossed with the two lines from which males were collected. Each cross was replicated 15 times.

All flies were live manipulated using aspirators for the remainder of the phenotypic assays to avoid any physiological and behavioral effects of CO_2_ anesthesia (Badre et al. 2005). Assays lasted 24 days and were conducted in two stages. During the first 10 days (i.e., first stage), female remating rates and egg-laying rates were measured; during the following 14 days (i.e., second stage), hatchability rates were measured.

In the first stage, females were transferred daily by aspirator into new vials with standard cornmeal fly food and blue food coloring. The dye helped visualize eggs laid by females without causing any variability in their behavior (Bergland et al. 2012). The vials also had 20 µL of a 10% diluted active yeast mixture to stimulate females’ reproductive activity. At lights on (i.e., dawn) on the initial day of the first stage, individual females were aspirated into a vial with two males from a single male line for mating. Approximately 90 min were allocated for copulation to occur, and all males were discarded immediately after this time period using an aspirator. Females that did not mate on the first day did not continue in the assay. Fecundity assays were conducted daily after the females were transferred into new vials. To assess short-term and long-term receptivity to remating effects, each individual female was introduced to two new males of the same genotype from her initial mating on the fourth and eighth day of the assay. Again we allowed 90 min on both remating days for copulations to occur, and all males were discarded via aspirator thereafter. Incorrectly sexed vials in which the female – instead of the male – was accidentally discarded were not included in later analysis.

Remaining vials that passed the first stage of the experiment were monitored daily for fly eclosion. Flies that eclosed were recorded and discarded immediately. Fly eclosion monitoring ended when either (1) three consecutive days of zero fly eclosions occurred or (2) 14 days of monitoring was reached – whichever occurred first. All phenotyping assays during the first and second stages were conducted within the first 3 h of lights on (i.e., dawn). All flies from the first stage and eclosing vials in the second stage were incubated at a controlled 25°C with a light timer set for a 12 h light:12 h dark regime.

### Longevity assays

Female flies used in our longevity assays come from (arranged from north to south) Selba, Alabama, USA (line 2-2); Thomasville, Georgia, USA (line 3-1); Freeport, Grand Bahamas-west (line 7-2); Bullock’s Harbor, Berry Islands (line 8-1); and Port Au Prince, Haiti (line 12-2). Representative “American” and “Caribbean” males were derived lines originating from the same male collection lines used in egg laying, hatchability, and remating assays, Birmingham, Alabama, USA (line 1-2), and Mayaguana, Mayaguana (line 11-1), respectively. “Homotypic” crosses were defined as male and female both of either American or Caribbean origin (i.e., American × American or Caribbean × American). “Heterotypic” crosses were defined as male and female from different origins (i.e., American × Caribbean or Caribbean × American). Males and females from the same origin were assumed to be more related and genetically similar to each other than those from different origins based on previous evidence (Yukilevich and True 2008b).

Virgin females were collected on light CO_2_ anesthesia and aged singly in vials for 4 days. Males were collected in the same manner and aged in groups of five per vial. We performed crosses in two separate rounds, which lasted approximately 70 and 80 days. In the first round, we crossed female flies from Selba, Alabama, USA, and Port Au Prince, Haiti, to either our representative “American” or “Caribbean” male. There were 50 replicates for each unique cross. Because of the large effect size from our initial round, we had 25 replicates for each type of cross in the rest of our lines. In each round, aged female flies were placed with five male flies for 48 h to ensure mating occurred. Male flies were discarded using an aspirator after the mating period. Female flies were then observed on a regular basis 5 days per week. Dates of deaths were recorded until the end of the 70- and 80-day observation periods. The females were transferred to fresh vials every 7 days.

### Postmating behavior data analysis

We examined the effects of geographic location on the total number of eggs laid by females, the total hatchability of those egg laid, and the propensity of females to remate 3 and 7 days after initial mating day. For egg laying and hatchability, we used ANOVA to test the effects of latitude and longitudinal coordinates (i.e., geographic position effects). We also used the male and female identity and phenotyping blocks to account for the variation from genotypes of male and females in addition to experimental block effects.

Because remating was scored as a binary variable of whether or not the female copulated on the two remating days, we used logistic regression models to assess the effects of geographic location while controlling for male and female genotypes and block effects on short- and long-term female receptivity to remating. The significance of longitudinal and latitudinal coordinates and model fits were assessed using analysis of deviance tables.

We performed a permutation test to investigate the significance of the lower hatchability rates in the three central locations as revealed by logistic regression models as well as through visual confirmation of plots. We calculated the difference in hatchability between the five lines from our three central locations and the hatchability of all other fly lines (18 lines). We then randomly assigned fly lines into groups of 5 and 18 and calculated the difference in hatchability between these two groups. These permutations were repeated 10,000 times. *P*-values were calculated by the number of times the difference in hatchability between these two groups were equal to or greater than our observed value divided by our 10,000 permutations. The line with the lowest hatchability was removed for a follow-up permutation test to confirm that the lower hatchability was only due to the effect of one line. Similar permutation tests were conducted on total egg counts to determine that lower hatchability was also not due to lower egg counts via ascertainment bias. Hatchability of eggs laid by females mated to representative “American” and “Caribbean” males were performed separately, and *P*-values from these tests were corrected using the Bonferroni method.

All analyses were performed in R, and the code for the permutation test is available upon request.

### Longevity data analysis

Survival analysis was used for temporal data of waiting times to an event with censored data. We employed methods from survival analysis to examine our data. We analyzed the waiting times of female death after homotypic or heterotypic mating. Females that escaped or survived past our observational periods were considered censored data points. The first step of survival analysis is to estimate survival functions for each of our crosses, S(*t*), which in our study is the probability of a female living longer than time, *t*. This can be carried out nonparametrically using the Kaplan–Meier method (Kleinbaum and Klein 2012). Parametric models were tested (i.e., exponential, lognormal, log-logistic, and generalized gamma), but none yielded a good fit (data not shown). After survival curves were fitted, we used it to estimate the cumulative hazard function, H(*t*), for each type of cross. The cumulative hazard function shows the cumulative probability that a female has expired up to time, *t*.

The most common statistical test used for comparing survival distributions is the log-rank test. However, this test has the proportional hazards assumption, which requires that the hazard functions of the two groups being compared are parallel. Hazard functions for our comparisons of female longevity after heterotypic and homotypic matings were plotted and visually checked for the crossing of hazard curves. When hazard curves cross, the proportional hazards assumption is violated, so another test must be conducted because the standard log-rank test has little to no power (Klein and Moeschberger 1997). We chose to use a combined weighted log-rank test, which takes into account crossing hazard curves (Bathke et al. 2005). This improved log-rank test has more power than the standard log-rank tests when the hazard functions cross and the hazard ratio is not proportional.

All analyses were performed in R using the “survival” package to estimate the survival curves and hazard functions. The package “emplik” was used as part of the improved log-rank test. The R code can be obtained online (http://www.ms.uky.edu/%7Emai/research/LogRank2006.pdf).

## Results

### Egg counts

**Figure 2:**
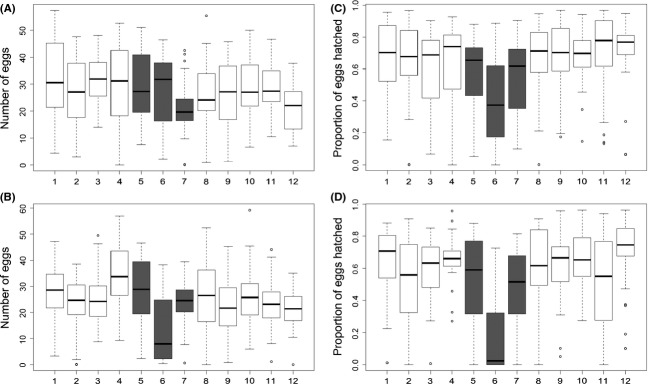
Egg counts of females mated with (A) American males and (B) Caribbean males. Hatchability of females mated with (C) American males and (D) Caribbean males. Each box plot is an isofemale line arranged from the northernmost location (left) to the southernmost location (right). Numbers on the *X*-axis correspond to those of Table1.

Egg counts do not appear to follow a clinal or other geographical pattern for females mated to representative “American” or “Caribbean” males. There is much variation among the lines, but the median egg count for each location is approximately the same except for when the females from location 6 (line 6-1; Sebastian, FL) were mated to Caribbean males (Fig.2B). The ANOVA model showed that most of the variance of egg laying was accounted for by male (*P* = 0.00167) and female (*P* < 0.0001) genotypes as well as block effects (*P* < 0.0001) and that longitude and latitude were not significant influences (*P* = 0.32767, *P* = 0.49860) (Appendix: Table4).

### Remating

Short-term remating rates were generally lower (range of rates: 0–30%) than long-term remating rates (range of rates: 0–60%). Remating rates do not appear to be influenced by location, which was investigated further with logistic regression.

The full logistic regression model evaluating effects of latitude and longitude while controlling for male and female genotypes and block effects found that latitude (*P* = 0.11) or longitude (*P* = 0.35) was not useful in predicting short-term remating rates or long-term remating rates (longitude *P* = 0.7616, latitude *P* = 0.6361). Male genotype was also not a significant influence on short-term or long-term remating rates (*P* = 0.4848 and *P* = 0.1240) (Appendix: Tables5, 7). The reduced models removing latitude and longitude as predictors showed that they were not significantly influencing remating rates (Appendix: Tables6, 8). Female identities in both logistic models for short- and long-term remating rates were significant, giving evidence that female genotypes could influence remating rates. However, when we fitted a model for long-term remating rates with a male × female interaction term, results showed that this interaction term was not significant (*P* = 0.0959) (Appendix: Tables9, 10).

### Hatchability

Hatchability in the three middle locations (location 4, 28, 33) at the border of the southeast United States and the Caribbean Islands appears lower than the locations on the edges in both the graphs displaying hatchability of females mated to American males (Fig.2C) and Caribbean males (Fig.2D). In the ANOVA model (Table2), longitude had a significant effect on hatchability (*F* = 3.954, *P* = 0.472) while latitude did not (*F* = 1.4, *P* = 0.2372) further suggesting that geographic location had some influence on hatchability rates as indicated by the dip in hatchability in Figure2C and D.

**Table 2:** ANOVA table for hatchability model

	DF	Sum Sq	Mean Sq	*F* value	*P*(>*F*)
Block	14	2.934	0.2096	4.688	<3.22e-08^*^
Female	22	7.722	0.3510	7.853	<2e-16^*^
Male	1	0.887	0.8869	19.844	9.84e-06^*^
Latitude	1	0.063	0.0626	1.400	0.2372
Longitude	1	0.177	0.1767	3.954	0.0472^*^
Female:Male	22	1.298	0.0590	1.320	0.1493
Residuals	672	30.035	0.0447		
Residuals	672	5,097,909			

From the permutation test to evaluate the significance of the dip in hatchability rates, we found that the hatchability in the middle three locations was significantly lower than the rates in the surrounding locations regardless of the female being mated to an American male (*P* < 0.0001) or Caribbean male (*P* < 0.0001). Results were similar when the location with the lowest hatchability rate was removed (28: Sebastian, FL, USA) and the permutation tests performed again (females mated to American male: *P* = 0.0056; females mated to Caribbean male: *P* = 0.0272). Similar tests were conducted on egg counts to investigate whether the lower hatchability was due to lower egg counts (i.e., ascertainment bias from not observing enough progeny). No significant differences in egg counts between females from the middle and outer locations were found regardless of whether they were mated to American males (*P* = 0.3192) or Caribbean males (*P* = 0.7584). Similar results were yielded when we removed the influence of the extremely low middle location, 28: Sebastian, FL, USA (mated to American males: *P* = 0.3016, mated to Caribbean males: *P* = 1.0). These results suggest a generalizable central location effect on hatchability.

### Longevity

**Figure 3:**
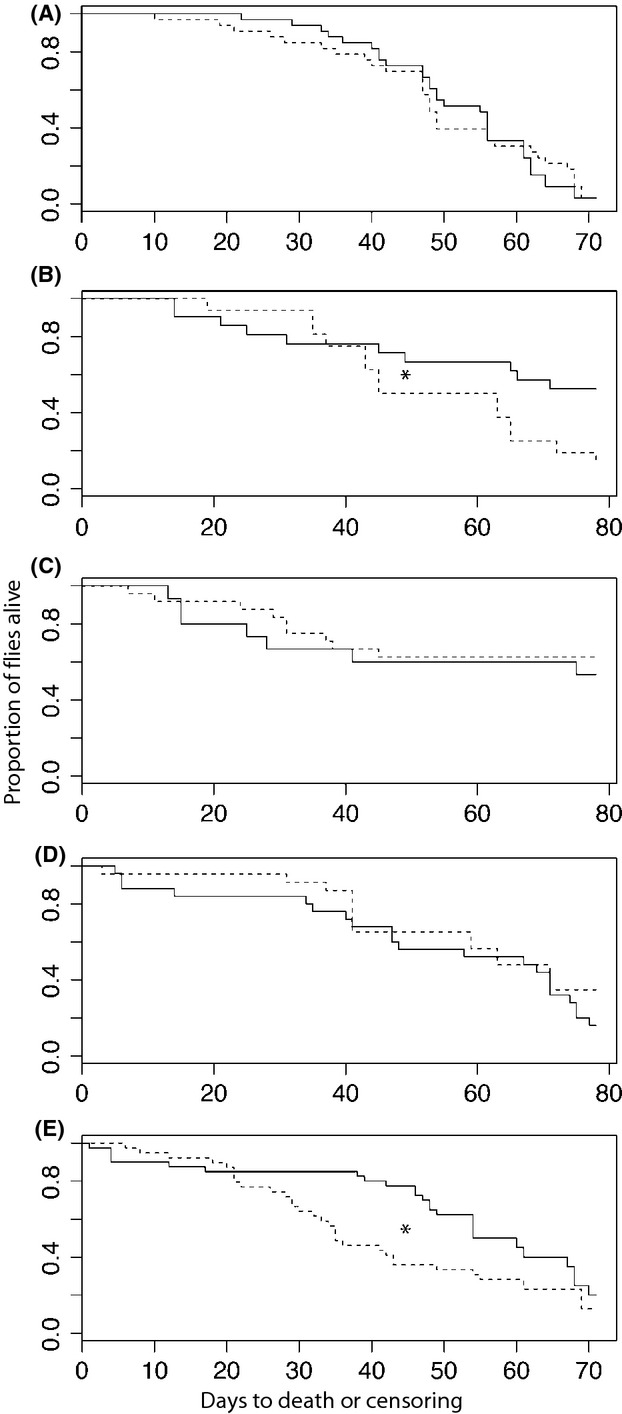
Survival curves of females of isofemale lines (A) 2-2, (B) 3-1, (C) 7-2, (D) 8-1, (E) 12-2 after experiencing homotypic (solid line) or heterotypic (dashed line) matings. *indicates significant *P*-value < 0.05.

The homotypic cross-survival curves for females from lines 2-2, 3-1, and 12-2 were consistently higher than the survival curves of females in heterotypic crosses (Appendix: Fig.3A, B and E). There were no apparent differences between homotypic and heterotypic survival curves of females originating from lines 7-2 or 8-1 (Fig.3C and D).

**Figure 6:**
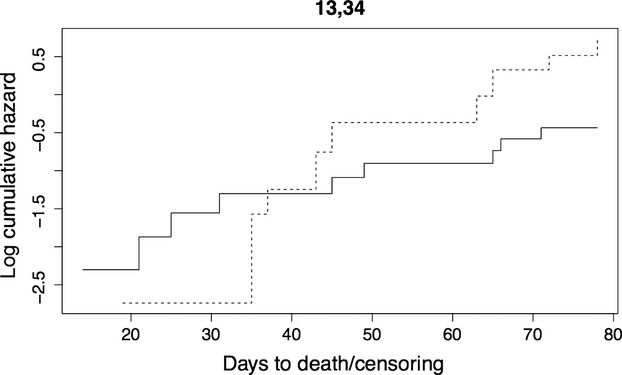
Hazard curves of females from line 13, 34 after experiencing homotypic (solid line) or heterotypic (dashed line) matings.

Hazard curves for all crosses and lines revealed nonproportional hazards in almost all cases of homotypic and heterotypic matings (Appendix: Fig.6). Crossing points of all hazard functions were visually estimated for use in the improved log-rank tests (Table3). The improved log-rank tests showed evidence that females after heterotypic matings had shorter life spans than females in homotypic matings for females from lines 3-1 and 12-2 (*P* = 0.0410 and *P* = 0.0271). Although females of line 2-2 visually displayed a reduced life span when involved in heterotypic matings (Appendix: Fig. 3A), these results were not significant in our statistical test (*P* = 0.3130).

**Table 3:** Improved log-rank test results

Isofemale line	T time of crossing hazards	*P*-value from improved log rank
3-1	37	0.04096407
8-1	42	0.4246727
7-2	40	0.6260448
12-2	23	0.02706502
2-2	61	0.3129819

## Discussion

We examined several potential postmating reproductive barriers including remating rates, egg-laying rates, hatchability, and female longevity that may potentially influence a system in the early stages of sexual isolation (Yukilevich and True 2008b). Our results illustrate the possible relationship between reproductive barriers and genetic admixture, and how admixture can shape geographical patterns of phenotypic characters.

### Genetic admixture likely affects offspring fitness

We observed a hatchability rate “valley” produced by isofemale lines originating from our three central locations spanning the border of the United States and the Caribbean Islands (i.e., locations 5, 6, 7). These locations correspond to areas of high African and European admixture (Kao et al. 2015). This result may highlight the presence of essential genetic differences between American and Caribbean fly populations, which could have manifested as an intrinsic postzygotic barrier between these populations. This type of evidence is indicative of the presence of Bateson–Dobzhansky–Muller incompatibilities (BDMIs), which are the most common form of intrinsic postzygotic isolation (Presgraves 2010). A reduction in the fitness of “hybrid” offspring here restricts the product of gene flow between American and Caribbean *D. melanogaster* populations. A more thorough investigation of these lines and genome sequences are required to confirm the presence of BDMIs, but are beyond the scope of this study.

### Females evolve resistance to toxic males

We examined female longevity after mating with males that were more or less genetically related to them. Degree of genetic relatedness was defined by geographical distance between the populations in which males and females originated from Kao et al. (2015). These results from the longevity assay were the inverse of our hatchability assays. Females originating from locations 7 and 8 did not seem as affected by heterotypic matings compared to females from the northern and southernmost locations (i.e., locations 2, 3, 12). It is known that male sperm has toxic effects on females after mating (Rice 1996) and, in response, females develop “resistance” against males that they coevolve with in the same environment (Arbuthnott et al. 2014). Our findings not only support this coevolution tactic but also illustrate that these patterns can occur in vivo.

## Conclusions

We did not find strong evidence that egg-laying rates or remating rates influence reproductive success in our system. However, this does not imply that egg-laying and remating receptivity are not influential postmating reproductive barriers, but, instead, may serve a role in other stages of speciation (Seehausen et al. 2014).

Overall, our data suggest that long-term postmating consequences – offspring fitness and female life span reduction – are of greater influence in this particular incipient sexual isolation scenario than more immediate postmating behavioral responses, such as egg-laying and remating receptivity. We have also observed possible effects of admixture at the border between the United States and the Caribbean islands (i.e., locations 5, 6, 7) (Kao et al. 2015) leading to interesting interactions between partially isolating mechanisms. Greater genetic admixture in flies originating from this area could promote the lower hatchability of eggs laid by females from these populations if American and Caribbean flies are genetically distinct enough to increase the possibility of DMIs occurring (Gompert et al. 2012). The same genetic admixture could also contribute toward female hardiness against harm from mating with a wider range of genetically diverse males, which in turn can compensate for lower hatchability by increasing reproductive life span.

Current views of speciation regard the process as a sliding continuum in which speciation can move forward or step back and may even be arrested at intermediate stages (Seehausen et al. 2014). Depending on the driving force of speciation, different types of reproductive barriers form at particular stages, with postmating barriers evolving at later stages of the speciation spectrum after premating barriers (Seehausen et al. 2014). The results from our study suggest that these postmating behaviors could be of importance earlier in the speciation continuum (Seehausen et al. 2014). Thus, speciation can take different paths with various types of reproductive barriers at any time in the process.

In summary, our study of postmating reproductive barriers along with previous investigations into premating barriers (Yukilevich and True 2008a,b) illustrates that pre- and postmating barriers could be evolving at the same time and are not necessarily sequential. While our findings contribute to the ever-growing breadth of knowledge about sexual isolation and speciation, it also sheds light on how isolating mechanisms evolve when genetic admixture is present.

## Figures and Tables

**Figure 4:**
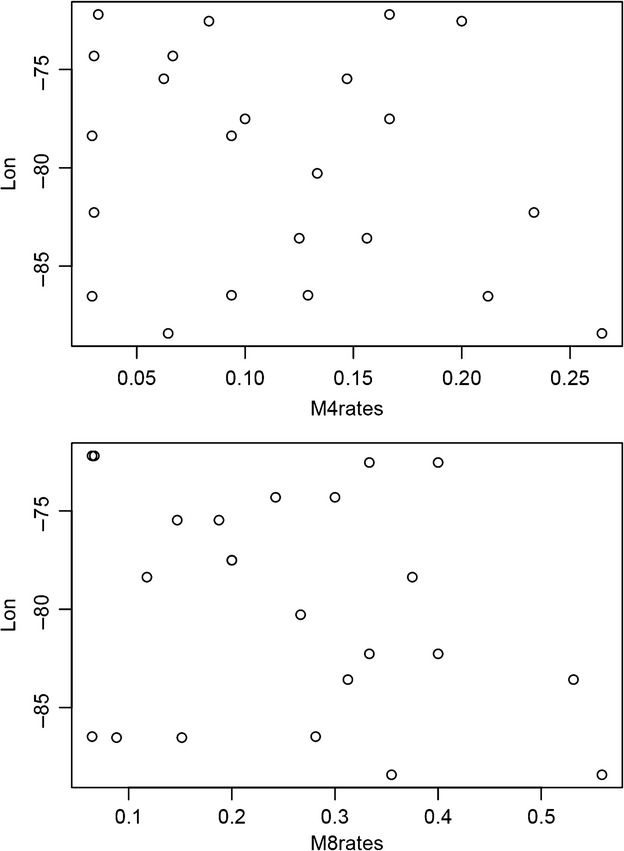
Short-term (top) and long-term (bottom) remating rates of females plotted against longitude.

**Figure 5:**
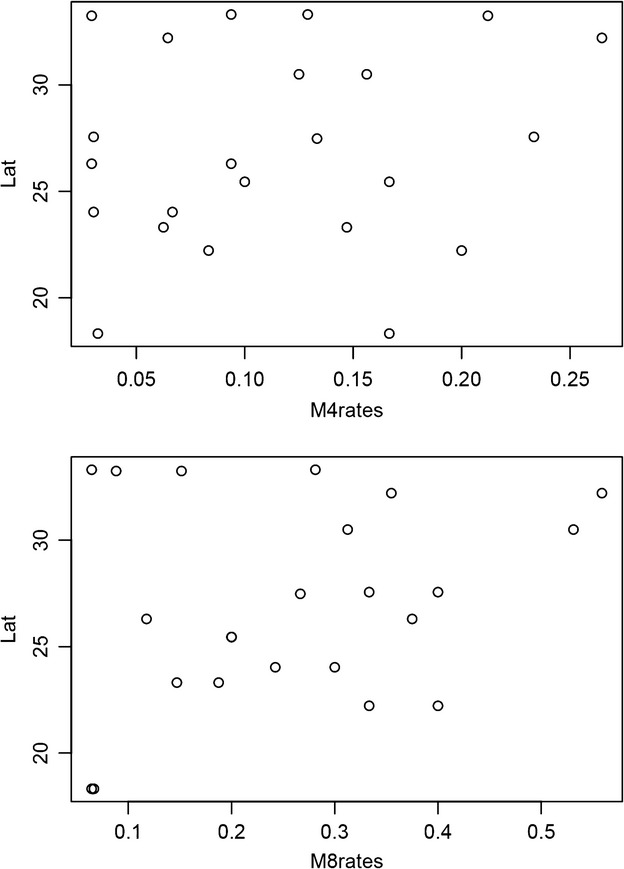
Short-term (top) and long-term (bottom) remating rates of females plotted against latitude.

**Figure 7:**
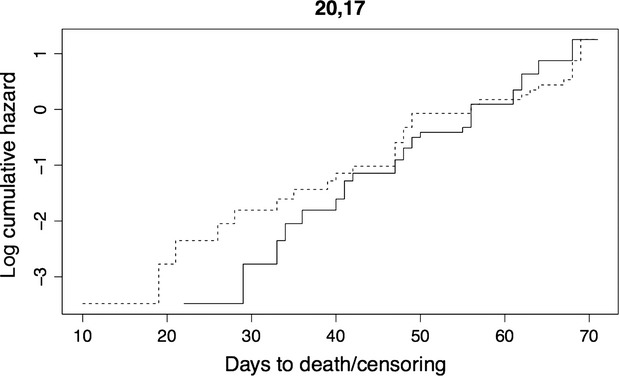
Hazard curves of females from line 20, 17 after experiencing homotypic (solid line) or heterotypic (dashed line) matings.

**Figure 8:**
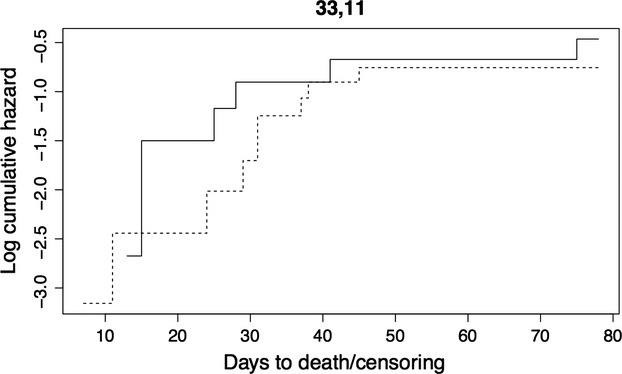
Hazard curves of females from line 33, 11 after experiencing homotypic (solid line) or heterotypic (dashed line) matings.

**Figure 9:**
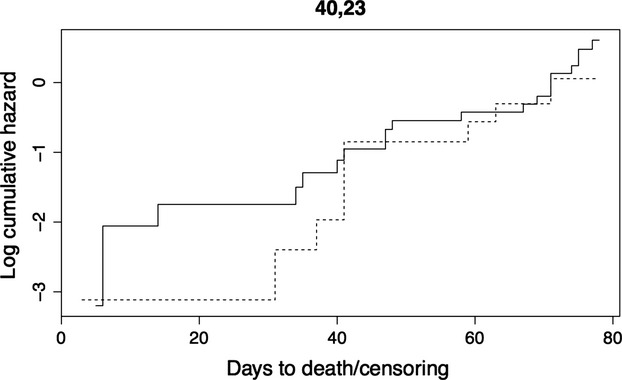
Hazard curves of females from line 40, 23 after experiencing homotypic (solid line) or heterotypic (dashed line) matings.

**Figure 10:**
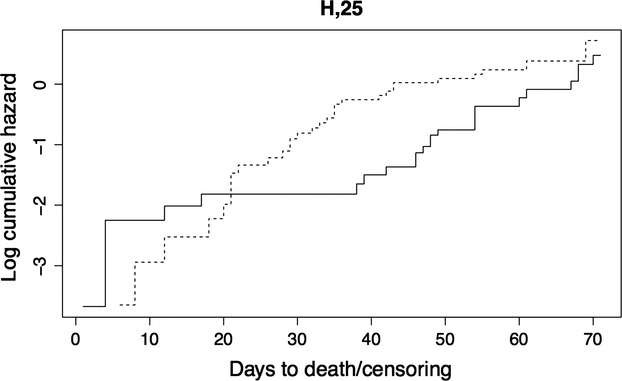
Hazard curves of females from line H, 25 after experiencing homotypic (solid line) or heterotypic (dashed line) matings.

## Appendix

**A1:** ANOVA results for average number of eggs laid

	DF	Sum Sq	Mean Sq	*F* value	*P*(>*F*)
Block	14	1,371,907	97,993	10.327	<2e-16^*^
Female	22	1,897,556	86,253	9.090	<2e-16^*^
Male	1	94,512	94,512	9.960	0.00167^*^
Latitude	1	9105	9105	0.959	0.32767
Longitude	1	4350	4350	0.458	0.49860
Female:Male	22	339,523	15,433	1.626	0.03539^*^
Residuals	672	6376,729	9489		

**A2:** Analysis of deviance table for full model of short-term remating rates and for reduced model of short-term remating without longitude or latitude

	DF	Deviance resid.	Df resid.	Dev	Pr(>Chi)
Full model					
NULL			733	522.17	
Female	22	35.152	711	487.02	0.037352
Male	1	0.488	710	486.53	0.484801
Block	14	30.945	696	455.59	0.005643
Latitude	1	2.602	695	452.98	0.106761
Longitude	1	0.865	694	452.12	0.352288
Reduced model					
NULL			733	522.17	
Female	22	35.152	711	487.02	0.037352
Male	1	0.488	710	486.53	0.484801
Block	14	30.945	696	455.59	0.005643

**A3:** Analysis of deviance table for short-term remating model comparison

	Res. Df.	Resid Df.	Df.	Dev.	Pr(>Chi)
Full	694	452.12			
Reduced	696	455.59	−2	−3.4667	0.1767

**A4:** Analysis of deviance table for full model of long-term remating rates and reduced model of long-term remating rates without longitude or latitude

	DF	Deviance Resid.	Df Resid.	Dev	Pr(>Chi)
Full model					
NULL			733	841.58	
Female	22	76.961	711	764.62	5.088e-08
Male	1	2.366	710	762.26	0.124014
Block	14	35.137	696	727.12	0.001403
Latitude	1	0.092	695	727.03	0.761588
Longitude	1	0.224	694	726.80	0.636064
Reduced model					
NULL			733	841.58	
Female	22	76.961	711	764.62	5.088e-08
Male	1	2.366	710	762.26	0.124014
Block	14	35.137	696	727.12	0.001403

**A5:** Analysis of deviance table for long-term remating model comparison

	Res. Df.	Resid Df.	Df.	Dev.	Pr(>Chi)
Full	694	726.80			
Reduced	696	727.12	−2	−0.31598	0.8539

**A6:** Analysis of deviance table for reduced model with female × male interaction term

	DF	Dev Resid	Df Resid	Dev	Pr(>Chi)
NULL			733	841.58	
Female	22	76.961	711	764.62	2.088e-08
Male	1	2.366	710	762.26	0.124014
Block	14	35.137	696	727.12	0.001403
MxF	22	31.012	674	696.11	0.095870

**A7:** Analysis of deviance table for reduced long-term remating model comparison with and without female × male interaction term

	Res. Df.	Resid Df.	Df.	Dev.	Pr(>Chi)
With FxM	674	696.11			
Reduced	696	727.12	−22	−31.012	0.09587
